# Neutral models of short-term microbiome dynamics with host subpopulation structure and migration limitation

**DOI:** 10.1186/s40168-018-0464-x

**Published:** 2018-04-27

**Authors:** Qinglong Zeng, Allen Rodrigo

**Affiliations:** 1 Department of Biology, Duke University, Durham, NC USA; 2 Research School of Biology, The Australian National University, Canberra, ACT Australia

**Keywords:** Microbiome, Diversity, Temporal dynamics, Metacommunity, Dispersal limitation

## Abstract

**Background:**

Most empirical studies tend to focus on microbiome dynamics within hosts or microbiome compositional differences between hosts over short periods. However, there is still a dearth of formal models that allow us to investigate the observed short-term dynamics of microbiomes under a unified ecological and evolutionary framework. In our previous study, we developed a computational agent-based neutral framework that simulates microbiome dynamics spanning many host generations with the added dimension of a genealogy of hosts. Although this long-term framework revealed interesting microbial diversity patterns under a simple but plausible evolutionary process and provided a platform for future elaboration of more complex systems, it does not allow us to explore microbiome dynamics within a single host generation.

**Methods:**

In this paper, we developed a computational, agent-based, forward-time framework of microbiome dynamics within a single host generation. As we have done under our neutral long-term models, we incorporate neutral processes of environmental microbiome assembly and microbe acquisition from parents and environment. We also incorporate a Moran genealogical model of hosts, so that the dynamics of microbiome evolution can be studied within a single host generation. Furthermore, we allow host subpopulation structure and host migration to affect microbiome recruitment.

**Results:**

We show that microbiome diversity within hosts increases monotonically with increases in environmental contribution, while microbiome diversity between hosts increases with increasing parental inheritance. Host population division and dispersal limitation under high host contribution further shaped the patterns by elevating microbiome differences between hosts and depressing microbial diversity within hosts. Microbiome diversity within the whole population showed strong temporal stability regardless of the modes of microbiome acquisition and subpopulation structures.

**Conclusions:**

We present a computational framework that integrates various processes including host genealogy, microbe recruitment, and host dispersal limitation acting on the short-term dynamics of microbiomes. Our framework demonstrates that the neutral dynamics of microbiomes within a population of hosts is strongly influenced by transmission mode and shared environment.

**Electronic supplementary material:**

The online version of this article (10.1186/s40168-018-0464-x) contains supplementary material, which is available to authorized users.

## Background

We previously developed a computational framework to model microbiome evolution spanning many host generations with the added dimension of a genealogy of hosts [1]. The framework allows us to investigate the effects of different ecological processes on microbiome diversity patterns over an evolutionary timescale measured on several thousand host generations. However, this long-term framework does not apply easily to most empirical studies, because these studies tend to focus on microbiome dynamics within hosts over short periods [2–4] or microbiome diversities within and between hosts [5, 6]. Many of these studies focus on microbial community establishment and succession within hosts [7–10], and responses to a variety of perturbations, including the administration of antibiotics [11–13], and changes in diet [14–16], lifestyle [17, 18], or environments [19, 20]. For host-associated microbial communities, therefore, it may be argued that the appropriate timescale for investigating microbiome dynamics is the lifespan of a host, or if we consider a population of hosts, a host generation. Over this period, microbes colonize and then proceed through several successional stages, frequently establishing a steady-state community (or, in ecological terms, a “climax” community) within the host, albeit one which can be altered if the host environment is disturbed [4, 21, 22]. In this paper, we develop a computational, agent-based, forward-time framework of microbiome dynamics within a single host generation to explore how microbial community composition responds under different conditions that affect recruitment and succession.

As we have done with the neutral models that we developed previously [1], we assume that microbial OTUs are functionally equivalent, and thus, do not differ in their fitness to survive in the host or environment. Nor do they confer any benefit to the fitness of their hosts. In other words, our models are selectively neutral. Unsurprisingly, this assumption of neutrality is often questioned. The association between host genetics and their microbiomes has been identified experimentally and statistically in several human and mouse studies [23–25]. Jerald et al. [26] also show that the evolutionary patterns in genomic data of gastrointestinal microbiomes can only be explained by niche processes despite the fact that empirical species abundance distributions fit the predictions of both neutral and non-neutral theories. Ley et al. argued that microbial diversity of host-associated microbiota is the result of entangled evolutionary forces on both microbe and host levels [27]. However, neutral models are commonly applied in model building: by constructing our models using simple assumptions, we are able to discern what patterns emerge in the absence of more complex processes, and thus compare these to real-world observations. We emphasize that we do not believe that host-microbe systems are as simple as our models, but only that it is more parsimonious to start with simple processes.

Thus, in the models presented in this paper, we also view recruitment either from the parent or from environment as a stochastic process that is influenced only by the relative abundance of each OTU at the source. Additionally, we allow microbial recruitment to be limited by subpopulation structure. With subpopulation structure, there is restricted exchange of hosts and microbes between subpopulations. Dispersal limitation of microbes under such conditions is a key component of metacommunity theory and is widely believed to be one of the most important ecological mechanisms that affect the assembly of microbial communities. Martiny et al. [28] suggest that geographic barriers and environmental heterogeneity are significant drivers of spatial variation in microbial diversity. Observations on free-living microbial communities [29–31] strengthen the claim that availability of microbes is often restricted by local environmental structures. Costello et al. [32] made a similar argument with respect to host-associated microbial communities by highlighting the role of dispersal limitation in mediating the diversity of human microbiota. Mihaljevic and others [33, 34] summarized the advantages and applications of linking a metacommunity model to symbiont communities. Other model-fitting studies utilized neutral metacommunity theory to explore the structuring mechanisms of human lung and parasitic helminth communities [35, 36].

By taking account of metacommunity structure and dispersal limitation, our framework views a host population as a collection of host subcommunities and their associated microbial communities, with exchanges of hosts and microbes that depend on the rates of host migration among communities: as hosts move between subpopulations, they carry their microbes with them and are able to pass these microbes on to offspring or the environment within their new subpopulation.

Our results indicate that in the absence of any subpopulation structure, parental contribution to microbial availability suppresses microbial diversity within individual hosts but increases the heterogeneity between hosts. We see the same pattern with our long-term models of neutral microbiome evolution [1]. In contrast, more complicated patterns of diversity are generated in the presence of host population substructure and migration. In particular, subdivided host populations promote local extinction of microbial taxa and inter-host differences in microbiome composition, although these effects are ameliorated by frequent host migrations.

## Models

**Fig. 1:**
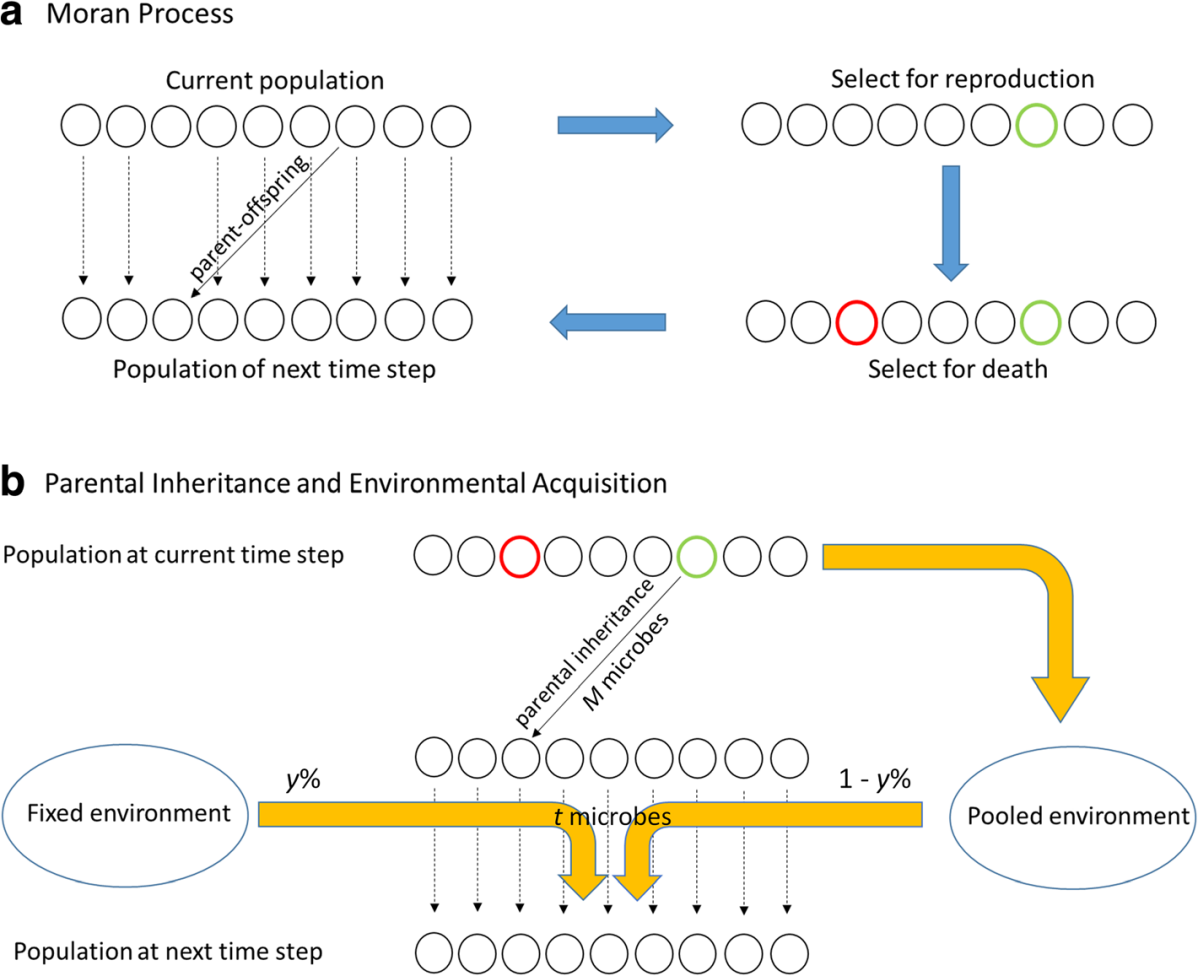
Microbiome parental inheritance and environmental acquisition under Moran process. (**a**) The diagram shows a population of hosts evolving under a standard Moran process, where the green circle indicates a dying host and the red circle indicates a reproducing host. The reproduced offspring will replace the dead one. *N* (where *N* is the population size) time steps of the abovementioned events are equivalent to one host generation. (**b**) The yellow arrows signify the flow of microbes from hosts to environment (pooled environment is a collection of microbes available in the current host population) and from environment to hosts (both fixed environment and pooled environment contribute to host microbiomes at next time step and the ratio of *y*% and 1-*y*% is determined by the environment assembly mode ME_*y*_). Parental inheritance of microbes only exists for newly reproduced offspring: *M* (the total number of microbes per host) microbes are inherited from parents at birth. All the hosts acquire *t* microbes from local environments at each time step: $t=\frac{aM}{N}$, where *M* is the total number of microbes per host, *N* is the host population size, *a* satisfies $x=\frac{1-{e}^{-a}}{a}$, and *x* is the expected percentage of parental microbes over one host generation determined by MA_*x*_ (see derivations in Methods)

Since our short-term models aim to investigate within-generation dynamics of host-associated microbial communities, a Moran genealogical model [37] is incorporated into the basic framework to simulate the stochastic changes in a host population of constant size with overlapping generations. A Moran model with host population size *N* has *N* time steps for one host generation and allows one random host reproduction and one random host death at each time step (Fig. 1a). As is described in Fig. 1b, this Moran genealogical process is included into the overall framework in a similar way as the Wright-Fisher process was incorporated under our previously proposed long-term neutral models of microbiome evolution, which allows us to model offspring microbiome acquisition and environmental microbiome assembly similarly as well (Fig. 1b).

We define the direct parental microbial contribution as the expected average percentage of microbes from the parent to its offspring over one host generation (i.e., over the *N* time steps). In our models, the single offspring that is produced at each time step obtains its microbiome by sampling exclusively from the microbiome of the parent. At each subsequent time step, some proportion of a host’s microbiome is sampled from the environment, and the rest is sampled from that individual’s “previous self” (i.e., that same individual’s microbiome in the preceding time step). In this way, a host’s microbiome is acquired as a mixture of parent and environmental microbial communities. We refer to this as a “mixed acquisition” process, indicated by MA_*x*_, where *x*% is the percentage of the microbiome recovered from the parent.

As with microbial acquisition, a mixture of processes determine the composition of the pool of microbes available from environment:

- A “fixed” environmental component (FE), whereby all microbial OTUs are available to hosts at every time step at an unchanging relative abundance.
- A “pooled” environmental component (PE), in which the microbial composition is determined by the relative abundance of OTUs in the total microbial pool from all hosts in the previous time step.
- A “mixed” environmental source (ME_*y*_) that contains a percentage, *y*%, from the parental pool of microbes, and (100-*y*)% of microbes from the fixed environment.

The acquisition and assembly of microbes in each host’s microbiome, and in the environmental pools, are driven by stochastic sampling under the assumption of ecological equivalence of hosts and microbes. The proportion of microbiome recruited from the host’s previous self is calculated as a function of parental contribution, so that over *N* time steps, the contribution at each time step will integrate to a total that is equal to the percent parental contribution per generation, *x*%. Unlike the direct contribution of a host to itself from one time step to the next, there is no need to integrate over all time steps for the hosts’ contribution to the environmental pool. This is because contributions from the host population in any given time step to the environment completely replace all previous contributions by hosts; thus, using a host contribution of *y*% to the environmental pool at each time step equates to *y*% host contribution over all *N* time steps.

**Fig. 2:**
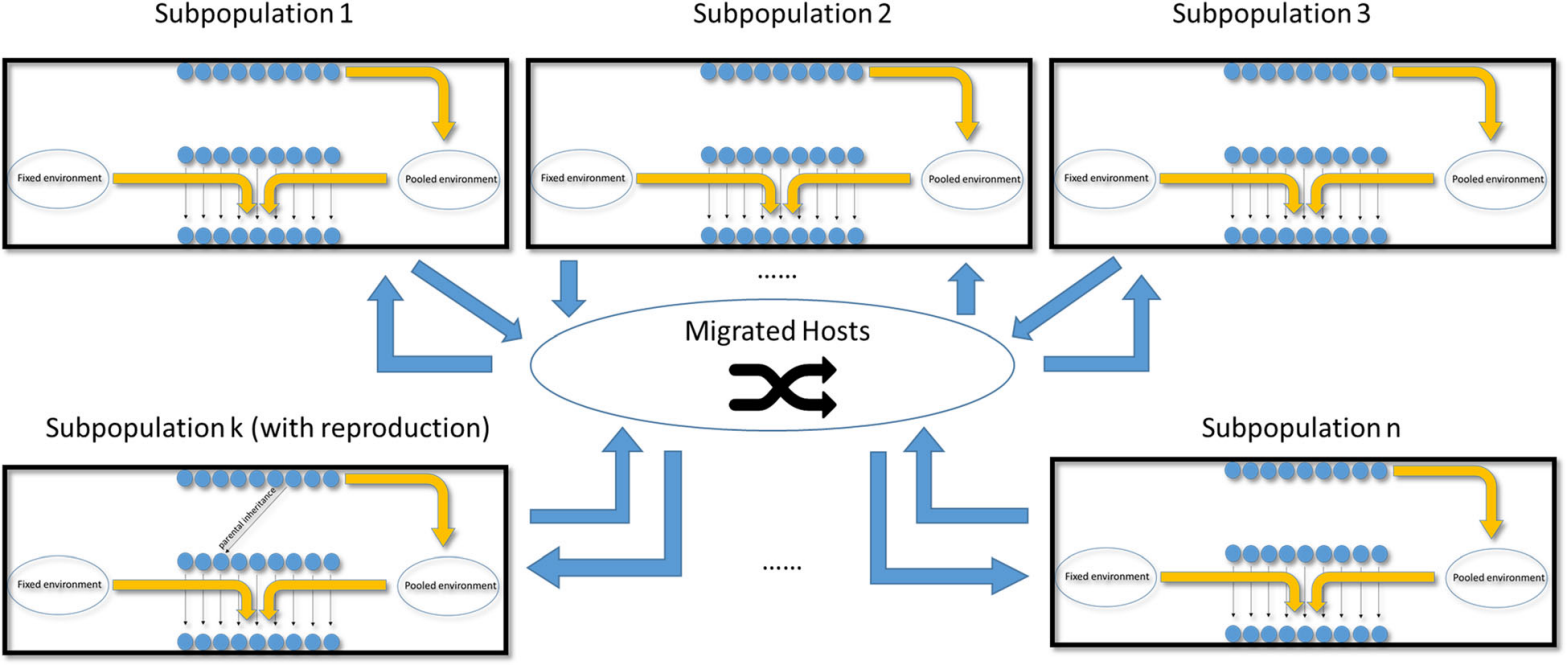
Population division and host migrations. Each black rectangle represents one host subpopulation. Each subpopulation undergoes the same process described in Fig. 1b except that only one subpopulation is randomly selected for host reproduction/death event at each time step. Host migration is a shuffling process. At the end of each time step, a percentage (determined by host dispersal rate) of hosts are randomly selected from all host subpopulations for migration. The original slots occupied by these migrating hosts are refilled by them in a shuffled order. If host migration rate is zero, no individuals are exchanged

Host population structure and dispersal limitation are incorporated after the basic platform is built (Fig. 2). With host population substructure, we allow a host population to be subdivided into several small subpopulations (often called “demes”; we use the two words interchangeably) with each subpopulation sharing a local environmental microbial community. Each local environment still consists of two parts: a fixed environmental (FE) component, which is a large multinomial sample from a common environmental microbiome, and a pooled environmental (PE) component, which is constituted by the microbiomes of hosts resident in a given subpopulation. The subpopulations may be completely isolated if no host migration is allowed; alternatively, with a non-zero host dispersal rate, a certain proportion of hosts in a subpopulation become “migrants.” All migrants are aggregated into a common pool, and then assigned randomly to each of the subpopulations. It is possible therefore that a “migrant” from a particular subpopulation is reassigned to that same population, although this probability decreases as the number of subpopulations increases.

The initial subpopulations of hosts (and their associated microbiomes) are constructed by performing progressive bifurcations of the host population, followed by independent resampling of microbiomes at each bifurcating node to generate independent instances of microbiomes for each subpopulation.

The Moran process allows us to model microbial flux within the host at a relative fine-grained timescale. For instance, in our simulations, we assume a metapopulation of 4096 hosts. Each generation is divided into 4096 time steps. If we apply our framework to a human population with an expected generation time of 30 years [38], then each time step corresponds to 64 h. In other words, our framework would allow hosts to recruit and exchange new microbes about every two-and-half days.

## Results

### Microbiome diversity dynamics in absence of subpopulation structure

**Fig. 3:**
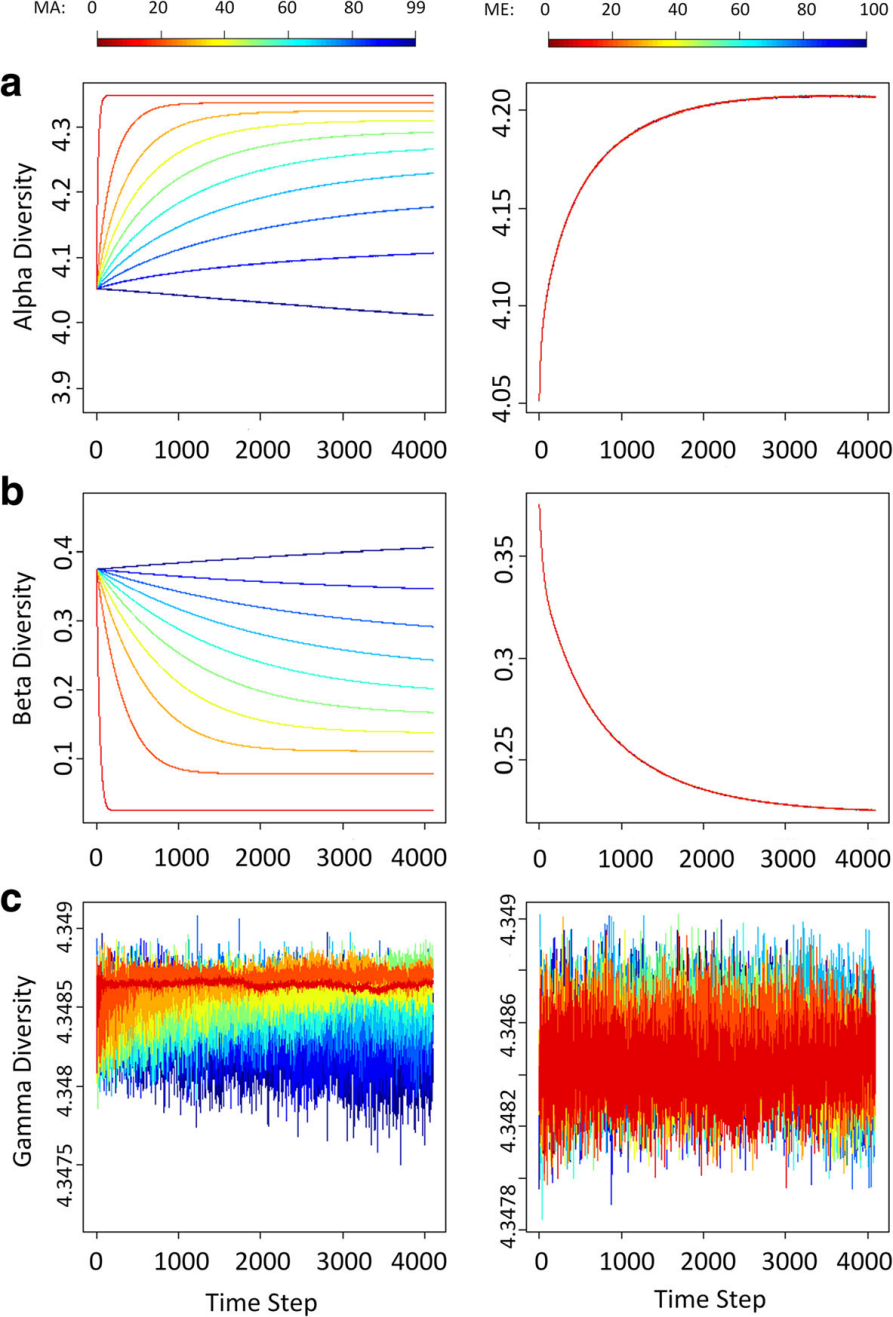
Diversity trace plots over time under different MA_*x*_ and ME_*y*_ in absence of population division. Each row labeled with (**a**), (**b**), or (**c**) represents one type of diversity (from top to bottom: *α*-, *β*-, and *γ*-diversities). Colors of lines indicate the percentage of parental inheritance (the left column: MA_*x*_) or the percentage of pooled environmental contribution (the right column: ME_*y*_)

At each time step of our simulations, we measured microbiome diversities within hosts (α-diversity) and within the whole population of hosts (*γ*-diversity) with the Shannon-Wiener Index and measured microbiome diversity between hosts (*β*-diversity) with the Bray-Curtis dissimilarity measure. In the absence of any population structure, our short-term simulations indicate that if there is high parental inheritance of microbiomes, hosts are apt to lose microbial species stochastically over time, resulting in low species diversity within hosts (*α*-diversity) and high differences between hosts (*β*-diversity) (Fig. 3b). In contrast, if microbiomes are obtained largely from the environment, a common environmental pool exerts a homogenizing effect, decreasing the difference between hosts (*β*-diversity) but enhancing the species richness and evenness within hosts (*α*-diversity) (Fig. 3a). Diversity across the whole population of hosts, *γ*-diversity, does not vary markedly over a single host generation regardless of how hosts acquire their microbes (Fig. 3c). Interestingly, the differences in parental contribution to a pooled environmental do not lead to significantly different values of *α*- and *β*-diversities in the absence of population structure (Fig. 3a, b).

Whereas the patterns of *α*-diversity obtained with our single-generation and long-term models are similar, the two models differ in the patterns obtained for *γ*-diversity and *β*-diversity [1]. In our long-term simulations [1], *γ*-diversity gradually declines instead of staying or fluctuating at the initial level, and *β*-diversity grows only for the first few generations and then declines under direct or indirect (via contributions to the environment) parental transmission. We think these differences between our short-term and long-term simulations result from the augmented ecological drifts at the level of the host population: the persistence and extinction of host lineages tend to intensify the demographic stochasticity in microbiomes over time and leads to the long-term depressive effects on *γ*- and *β*- diversities. This can only be captured by our long-term simulations, since it takes many host generations for a host population to lose a substantial number of lineages.

### Microbiome diversity patterns with subpopulation structure and host migration

**Fig. 4:**
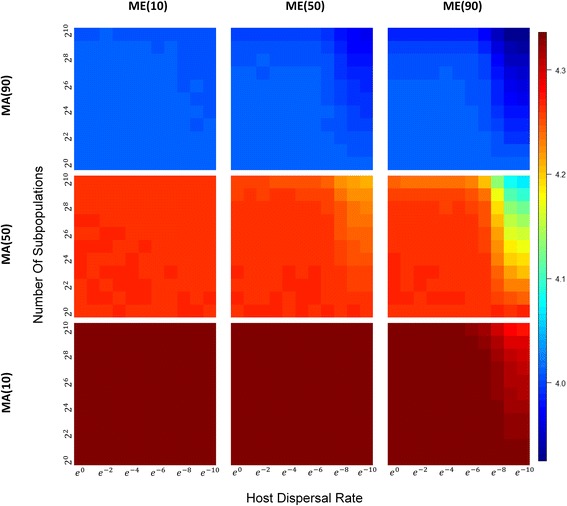
Heatmaps of α-diversity across a range of subpopulation sizes and host dispersal rate under different combinations of MA_*x*_ and ME_*y.*_ Each heatmap corresponds to a combination of MA_*x*_ and ME_*y*_ both of which has three levels (10%, 50%, and 90%). For each heatmap, horizontal and vertical axes represent the host dispersal rate and number of host subpopulations, respectively. The scales are logarithmic with range from 1 to 4096 for vertical axes and range from 1 to *e*^−10^ for horizontal axes. The color on the right of the heatmaps indicates the corresponding values for diversity (warm color: high diversity; cold color: low diversity)

With population subdivision, different patterns of *α*-, *β*-, and *γ*-diversities are obtained depending on the extent to which hosts contribute indirectly to the environment or directly to offspring, host migration rates, and the number of demes in the population (Table 1). When microbiomes are largely acquired from the environment (Fig. 4, bottom row), the number of demes and dispersal limitation show little effect on *α*-diversity, except at very low host migration rates. However, as parental contribution increases, either directly to offspring or indirectly through a contribution to the environment, we see that *α*-diversity decreases (Fig. 4), as the number of demes increase and the migration rate decreases.

**Table 1:** Summary of *α*-, *β*-, and *γ*-diversity patterns under different scenarios^a^

	Number of subdivision	Low number of demes		High number of demes	
		High migration rate	Low migration rate	High migration rate	Low migration rate
High parental contribution	*α* ↓ ↓ *β* ↑ ↑ *γ*~	*α* ↓ ↓ *β* ↑ ↑ *β*_*w*_ ↑ ↑ *β*_*b*_ ↑ ↑ *γ*~ *γ*_*w*_~	*α* ↓ ↓ ↓ *β* ↑ ↑ ↑ *β*_*w*_ ↑ ↑ *β*_*b*_ ↑ ↑ ↑ *γ*~ *γ*_*w*_↓	*α* ↓ ↓ ↓ *β* ↑ ↑ ↑ *β*_*w*_ ↑ ↑ *β*_*b*_ ↑ ↑ ↑ *γ*~ *γ*_*w*_ ↓ ↓ ↓	*α* ↓ ↓ ↓ ↓ *β* ↑ ↑ ↑ ↑ *β*_*w*_↑ *β*_*b*_ ↑ ↑ ↑ ↑ *γ*~ *γ*_*w*_ ↓ ↓ ↓
High environmental contribution	*α* ↑ ↑ *β* ↓ ↓ *γ*~	*α* ↑ ↑ *β* ↓ ↓ *β*_*w*_ ↓ ↓ *β*_*b*_ ↓ ↓ *γ*~ *γ*_*w*_~	*α* ↑ ↑ *β* ↓ ↓ *β*_*w*_ ↓ ↓ *β*_*b*_ ↓ ↓ *γ*~ *γ*_*w*_~	*α* ↑ ↑ *β* ↓ ↓ *β*_*w*_ ↓ ↓ *β*_*b*_ ↓ ↓ *γ*~ *γ*_*w*_ ↓ ↓ ↓	*α* ↑ ↑ *β* ↓ ↓ *β*_*w*_ ↓ ↓ *β*_*b*_ ↓ ↓ *γ*~ *γ*_*w*_ ↓ ↓ ↓
High parental contribution (long term)	*α* ↓ ↓ *β* ↑ ↓ *γ* ↓ ↓	–	–	–	–
High environmental contribution (long term)	*α* ↑ ↑ *β* ↓ ↓ *γ* ↑ ↑	–	–	–	–

^a^↓: declining; ↑: increasing; more consecutive ↓/↑ represents a greater change; ↑↓:first increasing then declining; ~: fluctuation and no obvious trend

**Fig. 5:**
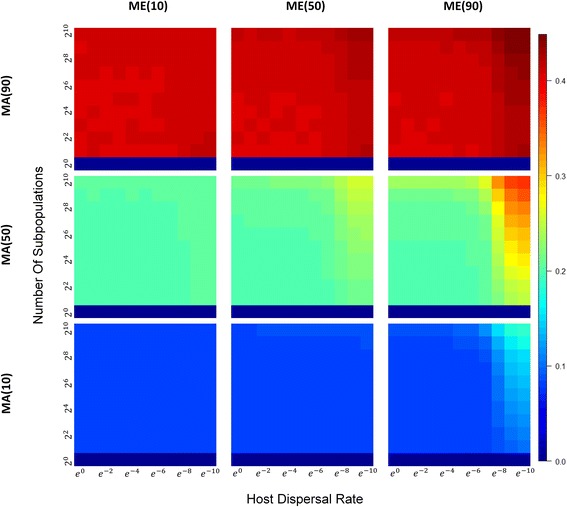
Heatmaps of *β*_*b*_-diversity across a range of subpopulation sizes and host dispersal rates under different combinations of MA_*x*_ and ME_*y.*_ With a similar layout, all heatmaps are also plotted in the same way as those in Fig. 4 except that *β*-diversity between subpopulations instead of overall *β*-diversity is measured

**Fig. 6:**
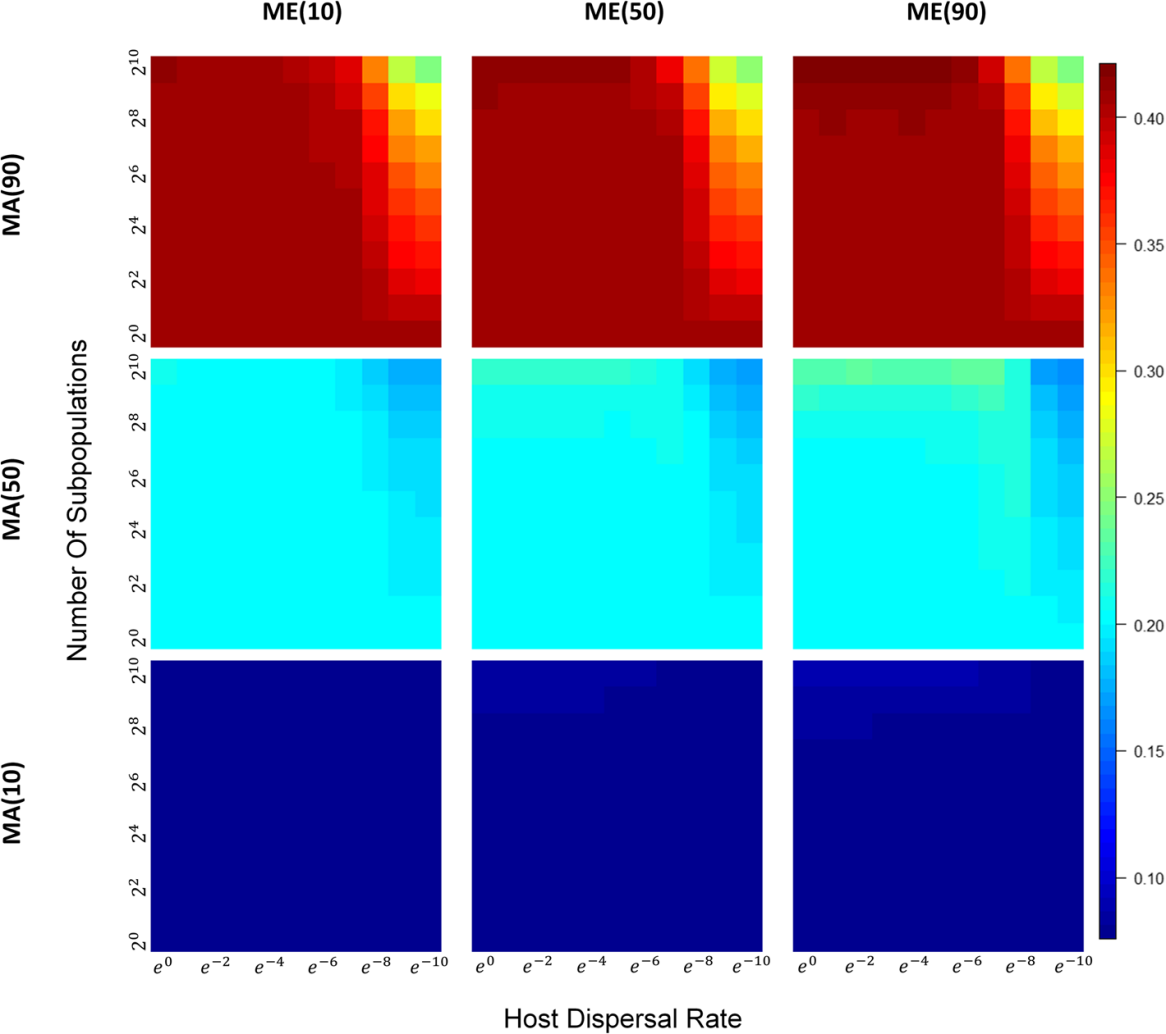
Heatmaps of *β*_*w*_-diversity across a range of subpopulation sizes and host dispersal rates under different combinations of MA_*x*_ and ME_*y.*_ With a similar layout, all heatmaps are also plotted in the same way as those in Fig. 4 except that *β*-diversity within subpopulations instead of overall *β*-diversity is measured

**Fig. 7:**
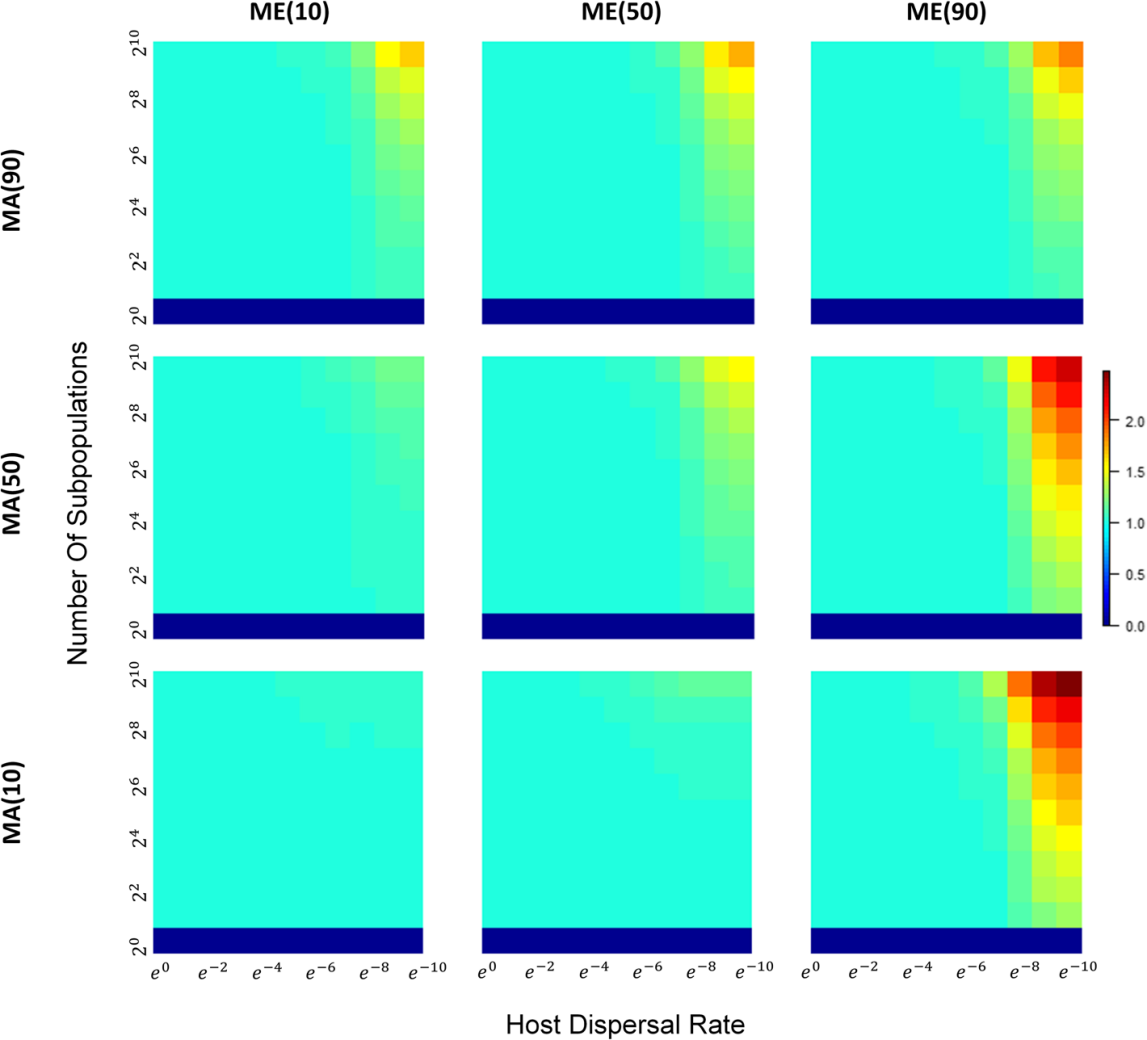
Heatmaps of ratio of *β*_*b*_-diversity to *β*_*w*_-diversity across a range subpopulation sizes and host dispersal rates under different combinations of MA_*x*_ and ME_*y.*_ With a similar layout, all heatmaps are also plotted in the same way as those in Fig. 4 except that *β*-diversity ratio instead of *α*-diversity is measured

We see the converse with between-host diversity, both for hosts between demes (i.e., *β*_*b*_-diversity; Fig. 5), and within demes (*β*_*w*_-diversity; Fig. 6): when direct or indirect parental contribution is low and between-host diversity is low. Interestingly, if we compare *β*_*b*_-diversity to *β*_*w*_-diversity (Fig. 7), we see that as the number of demes increase, and host migrations decrease, differences between hosts in different demes is greater than the differences between hosts in the same deme. This makes sense, of course, because a highly divided population under limited host migration is equivalent to having many “islands” of small isolated subpopulations. *β*_*w*_-diversity tend to decrease as small subpopulations are more apt to be homogenized by a shared environment; *β*_*b*_-diversity tend to be elevated because subpopulation structure and isolation allow each subpopulation to evolve with greater independence and stochastic differences between subpopulations gradually accumulate over time.

What about microbial *γ*-diversity of the collection of hosts across all subpopulations, i.e., the host metapopulation? As opposed to changes that may occur in the environmental microbial component within demes, *γ*-diversity across the metapopulation is not affected by population subdivision (Additional file 1: Figure S1). In retrospect, perhaps this is unsurprising: under a neutral model of microbiome dynamics, a single host generation does not appear to be able to alter microbial diversity across the entire population. However, as the number of demes increases, and migration rates decrease, within-deme *γ*_*w*_-diversity decreases (Additional file 2: Figure S2). This is because microbial species carried by migrants diversify local communities and individual microbiomes. As the numbers of migrants decrease, there is less opportunity to homogenize the microbial composition in different demes, although metapopulation microbial diversity is maintained. Therefore, it is possible to see microbial *γ*-diversity of the metacommunity of hosts remaining relatively stable for different degrees of population fragmentation but decreasing within each subpopulation.

## Discussion

In this paper, we introduce an agent-based computational framework that implements short-term neutral models of microbiome dynamics using a Moran process. The framework presented here has many points of similarity with the long-term computational framework developed earlier [1], including the application of different acquisition and environmental assembly modes. However, unlike the Wright-Fisher process that was used in our long-term framework, the Moran process allows us to investigate the temporal dynamics and diversity patterns of microbiomes within a single host generation. We have included host dispersal and subpopulation structure in our short-term models to help us explore the effects of dispersal limitation on microbiome diversities. Empirically, these subpopulations may correspond to different host communities, locales, or family groups. Although our short-term models do not include non-random shifts in microbial composition and/or host dynamics caused by dietary or environmental changes, illness, antimicrobial therapies, etc., it is theoretically easy to incorporate these types of events into the Moran model, because we are able to perturb the dynamics of the system at discrete time steps. Adding these extensions to our framework is an obvious next step.

Under our short-term framework, each host’s first microbes are acquired from parents at birth (vertical transmission). The seeding effects of early exposure to maternal environments on initial microbial communities have been studied for humans and other mammals [39–41]. Postnatal microbial succession is abstracted in our models via a constant process of microbial recruitment from a host’s previous self (i.e., the same host in the previous time step) and the environment (horizontal transmission). In fact, as hosts age, both human and plant examples [4, 42, 43] show a successional trend in individual microbiomes from low diversity to high diversity gradually reaching a steady state, most likely a consequence of persistent microbial colonization from the environment. Our simulations here also recover the same trend (Additional file 3: Figure S3). Varying the proportion of parental contribution and environmental contribution under the short-term models has similar impacts on *α*- and *β*-diversities as they do with our the long-term models (Table 1) [1], although population subdivision appears to intensify these effects. Similar patterns were also observed in some natural communities; for instance, cnidarian hosts that acquire their algal symbionts horizontally appear to harbor more diverse communities than hosts that acquire their symbionts vertically [44].

A point of contrast between our short-term and long-term models was observed with *γ*-diversity, with the long-term models having lower levels of whole-population diversity relative to our short-term models. We believe that this is a consequence of imposing a Wright-Fisher genealogical model on our long-term simulations. Under such a genealogy, we expect that after a sufficient number of generations have elapsed (on average 2 *N* generations, for haploid hosts, or 4 *N*, for diploid hosts), all individuals will be descended from a common ancestor. Depending on the rates of parental contribution to the environment, we are likely to see greater loss of whole-population microbial diversity in long-term models, relative to our short-term models.

Our short-term models also include the effects of dispersal limitation in a structured host population, and they allow us to recover the patterns of diversity shaped by shared environments that we see in real-world situations. For example, a comparative study on the human gut microbiome indicated significant species diversity differences between a farming group and an urban control group as well as between males and females in the farming group [45]. The uncommon sexual differentiation of gut microbiomes in Hadza is believed to be associated with the sexual division of labor and diet in this special hunter-gatherer community. This marked heterogeneity between different social groups provides empirical evidence for the predictions of our models about population division and shared environment on *β*-diversity. Similarly, more skin and oral microbiota were found to be shared between cohabiting adults than with other individuals regardless of host genetic relatedness [46–48]. These interesting observations are consistent with our predictions of increased microbiome similarity within-host subpopulations and decreased microbiome similarity between host subpopulations (Figs. 5, 6, and 7) and highlight the dominant role of shared environments in determining microbiome compositions that contrast with the effects of selective factors from host genetics.

To summarize, in this paper, we present a computational framework to model the host-microbe dynamics with and without population subdivision, over a single host generation. As with our previously proposed long-term framework [1], we model microbiome dynamics within a host population and consider a shared evolutionary history between hosts and microbes by simulating host genealogy. Our results confirm what theory tells us to expect: as we increase the number of subpopulations and decrease the rate of host exchanges between the subpopulations, we see decreasing levels of within-host diversity and increasing levels of between-host diversity [49]. Both are likely consequences of the increasing effects of ecological drift acting on microbial communities from local environmental and/or host communities. The incorporation of host subpopulation structure and dispersal limitation also highlights the significant effects of shared environment in shaping core microbiome within subpopulations in the absence of selection.

But if our models confirm what community microbial theory predicts, what value do these models have? We believe that the value of our computational models resides in the assumptions and processes that underlie the models’ framework. By stating explicitly what these assumptions and processes are, our models can be examined and modified. In effect, our models, and others like them, serve as computational “proofs” of microbial ecological theory. And by making our models as simple as possible, they also serve as “null hypotheses” for statistical tests, allowing researchers to perform forward neutral simulations using their own empirical microbiome data as the initial microbiomes to forecast short-term dynamics under custom settings of parental/environment contribution and host population structures.

The Moran model used here is also particularly flexible, allowing users to intercept simulated populations at any time step to accommodate transient or sustained changes in hosts, microbes, and environments. It is also possible to extend the framework to include the effects of selection acting on hosts and/or microbes, as we have done recently [50]. As we noted above, the important role of non-neutral effects in microbiome assembly has been investigated and demonstrated by others [25, 27, 51]. We expect the incorporation of selection into our current framework may further drive the differentiation of microbiomes between host subpopulations and facilitate the establishment of core microbiomes within each deme.

## Conclusions

Previous empirical studies suggest a need for ecological and evolutionary frameworks to investigate short-term dynamics of microbiomes. Here we present a computational framework that incorporates models that take account of multiple ecological or evolutionary processes including host genealogy, microbe acquisition, environmental microbiome assembly, host subpopulation structures, and migration to model microbiome dynamics within a single host generation. Our simulated results indicate that high parental inheritance raised microbiome differences between hosts, while high environmental contribution increased microbiome diversity within hosts. Our framework also shows that host subpopulation structure and dispersal limitation can further reshape the diversity patterns when host contribution to offspring microbiome is high. Highly fragmented host population with limited dispersal rate increases the overall *β*-diversity and *β*-diversity between subpopulations and decreases *β*-diversity within subpopulations and *α*-diversity.

## Methods

As mentioned before, we implemented our short-term simulations in a similar way as we did for our long-term neutral framework. The simulated host population has a constant size of *N* hosts (*N* = 4096). Each host is allocated a virtual microbiome with capacity of *M* microbes (*M* = 10^7^). In order to initialize the simulations, we obtained empirical human microbiome data at the genus level from HMP website (https://www.hmpdacc.org/HMSMCP/). The initial microbiomes are resampled from a truncated and rescaled multivariate normal distribution (trMVN) which is fitted to the empirical microbiome data from human stool samples [52]. A Kolmogorov-Smirnov test demonstrates that the resampled microbiome data still maintains the same microbial abundance and diversity distributions of empirical data.

For the sake of simplicity, we have the same number of hosts in each subpopulation (the methods can be modified to allow different subpopulation sizes to be simulated). As noted above, the total number of hosts is 4096 = 2^12^. To construct *k* subpopulations, we construct a complete binary tree with *k* levels. For example, when *k* = 1, there is a root node on the tree, and two terminal nodes, representing the two host subpopulations. The microbiome at the root node is described by the trMVN fitted with empirical data. The microbiomes at any node that represents a subpopulation are also described by a trMVN drawn from the trMVN of its parental node. The hosts within each subpopulation represent random draws from the microbiome of the subpopulation. In this way, we are able to generate host microbiomes in 2^*k*^ equally sized subpopulations, where *k* ∈ [*ℤ*; 0, *d*].

The fixed environmental microbiome was simulated by pooling all empirical human stool microbiome data. Thus, the total number of OTUs we simulated is the total number of genera contained in this empirical human microbiome dataset (*g* = 129). Of course, simulating less or more OTUs at higher or lower taxonomical ranks is also possible. It is plausible to conjecture that increasing the total number of OTUs will be expected to reduce the average population size per microbial taxon and thus increase the sensitivity of microbiomes to the effect of ecological drifts since rare OTUs are more likely to be affected by demographic stochasticity [53–55]. Therefore, under high host contribution with/without subpopulation structures, *α*-diversity may decrease further and *β*-diversity may increase further. We do not believe that this will alter the qualitative results that we have obtained.

Under the scenario of ME_*y*_, environmental microbiomes $\overrightarrow{e}$ are updated at each time step by mixing two environmental components as follows:

$$ \overrightarrow{e}=\left(1-y\right)\overrightarrow{f}+y\overrightarrow{p,} $$

where $\overrightarrow{f}$ and $\overrightarrow{p}$ represent the fixed environment and pooled environment, respectively. As noted above, the pooled environmental microbiomes $\overrightarrow{p}$ are constructed by pooling all the microbes in the current host populations if no host population structure is simulated, or in the corresponding host subpopulations when the host population is segmented:

$$ \overrightarrow{p}=\frac{1}{n}\sum \limits_{i=1}^n{\overrightarrow{a}}_i, $$

where ${\overrightarrow{a}}_i$ represent the microbiome composition of host *i* in current subpopulation/population of size *n*.

Under the scenario of MA_*x*_, the newborn offspring always inherits their initial microbiome (10^7^ microbes) from the parent and then continues to acquire microbes at a constant rate (*v* microbes per time step) from the environment. Let *T* be the total number of microbes acquired from the environment within one host generation; at each time step, one host receives *t* = *T*/*N* microbes from the environment. Let *M* denote the total number of microbes in one microbiome. The expected average percentage of parental microbes over one host generation, *x*, can be written as follows:

$$ x=\frac{1}{N}\sum \limits_{i=0}^{N-1}{\left(\frac{M}{M+t}\right)}^i\approx \underset{N\to \infty }{\lim}\frac{1}{N}\sum \limits_{i=0}^{N-1}{\left(\frac{M}{M+t}\right)}^i=\frac{1-{e}^{-a}}{a}, $$

where $a=\frac{T}{M}=\frac{Nt}{M}$. By solving the abovementioned equation, we can find out the value of *a* corresponding to any parental contribution; this gives us the number of microbes each host acquires from the environment at one time step for the implementation of simulations. Since our long-term models also interpret parental contribution as the expected percentage of parental microbes over one host generation, the parental contribution *x* defined above is equivalent with the parental contribution parameter defined under our long-term models.

As is mentioned before, the initial host population structure is formed by performing progressive bifurcation of the host population and hierarchical clustering of the initial microbiomes. Each cluster of host microbiomes represents an initial host subpopulation. At each time step, we randomly select one host subpopulation for the event of host reproduction and death. Then, we draw *z*_*j*_ hosts from host subpopulation *j* as migrating hosts with *z*_*j*_~*Bin*(*n*_*j*_, *q*). *Bin*(*n*_*j*_, *q*) represents a binomial distribution, where *n*_*j*_ is the size of subpopulation *j* and *q* is the host dispersal rate. We shuffle all the migrating hosts from the whole population and randomly reassign them back to their new subpopulations.

Three diversities are measured by the Shannon-Wiener index (*α*- and *γ*-diversities) and the Bray-Curtis dissimilarity index (*β*-diversity). The Shannon-Wiener index is a popular diversity index in ecological studies and quantifies both species richness and evenness in a community while the Bray-Curtis index is a non-Euclidean distance measurement and often used to quantify the compositional dissimilarity between two sites. More specifically, we have three types of *β*-diversities: overall, between-subpopulation, and within-subpopulation. For overall *β*-diversity, we did a pairwise comparison to compute the average microbiome difference between hosts. For between-subpopulation/within-subpopulation *β*-diversity, we only considered host pairs from two different subpopulations/from the same subpopulation. We also have measured *γ*-diversity within subpopulation by averaging *γ*-diversities of all subpopulations.

## Additional files

Additional file 1: **Figure S1.** Heatmaps of *γ*-diversity across a range subpopulation sizes and host dispersal rates under different combinations of MA_*x*_ and ME_*y.*_ With a similar layout, all heatmaps are also plotted in the same way as those in Fig. 4 except that *γ*-diversity instead of α-diversity is measured. (TIF 3353 kb)

Additional file 2: **Figure S2.** Heatmaps of average *γ*_*w*_-diversity across a range subpopulation sizes and host dispersal rates under different combinations of MA_*x*_ and ME_*y.*_ With a similar layout, all heatmaps are also plotted in the same way as those in Fig. 4 except that *γ*_*w*_-diversity within subpopulations instead of α-diversity is measured. (TIF 1286 kb)

Additional file 3: **Figure S3.** *α*-diversity trace plots of individual microbiomes over host lifespan. Individual within-host diversity plots show under both structured population (1024 demes and host migration rate = *e*^*− 10*^) and unstructured population, individual *α*-diversity increases along time after birth. The blue lines represent hosts who are still alive before our simulation ends. The red lines represent hosts whose death events are observed by the end of our simulation. (TIF 5367 kb)

## Abbreviations

MA_*x*_: Mixed acquisition with *x*% from parental inheritance and (1-*x*)% from environment
ME_*y*_: Mixed environmental microbiome with *y*% from pooled environment and (1-*y*)% from fixed environment
