# Supplementary figures and images for: Neutral models of short-term microbiome dynamics with host subpopulation structure and migration limitation
Source: Microbiome. 2018 Apr 27;6:80. doi: 10.1186/s40168-018-0464-x (PMC5921780; doi:10.1186/s40168-018-0464-x)

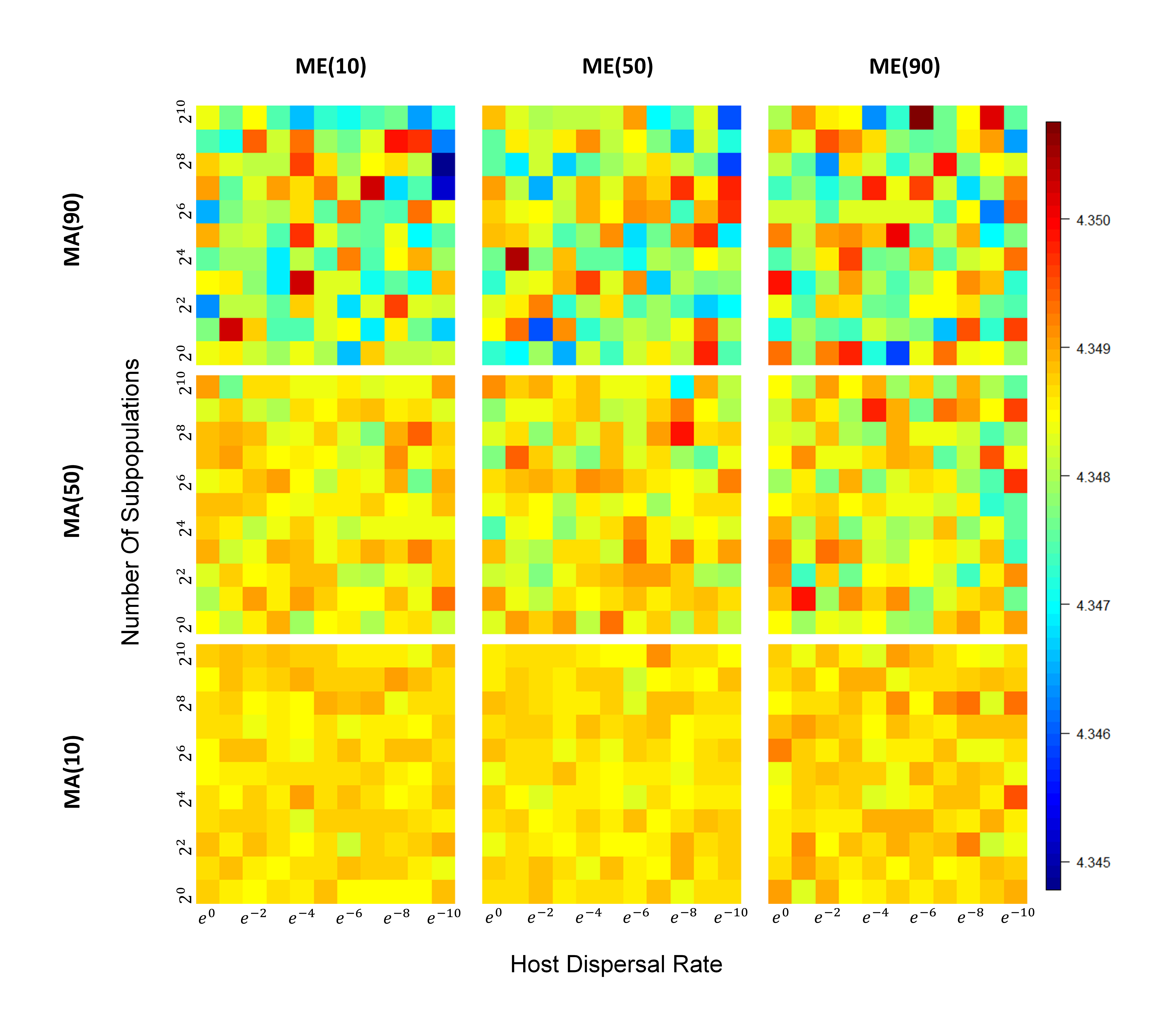

Supplement: Supplementary file 1 — Figure S1. Heatmaps of γ-diversity across a range subpopulation sizes and host dispersal rates under different combinations of MAx and MEy. With a similar layout, all heatmaps are also plotted in the same way as those in Fig. 4 except that γ-diversity instead of α-diversity is measured. (TIF 3353 kb) [file 40168_2018_464_MOESM1_ESM.tif]

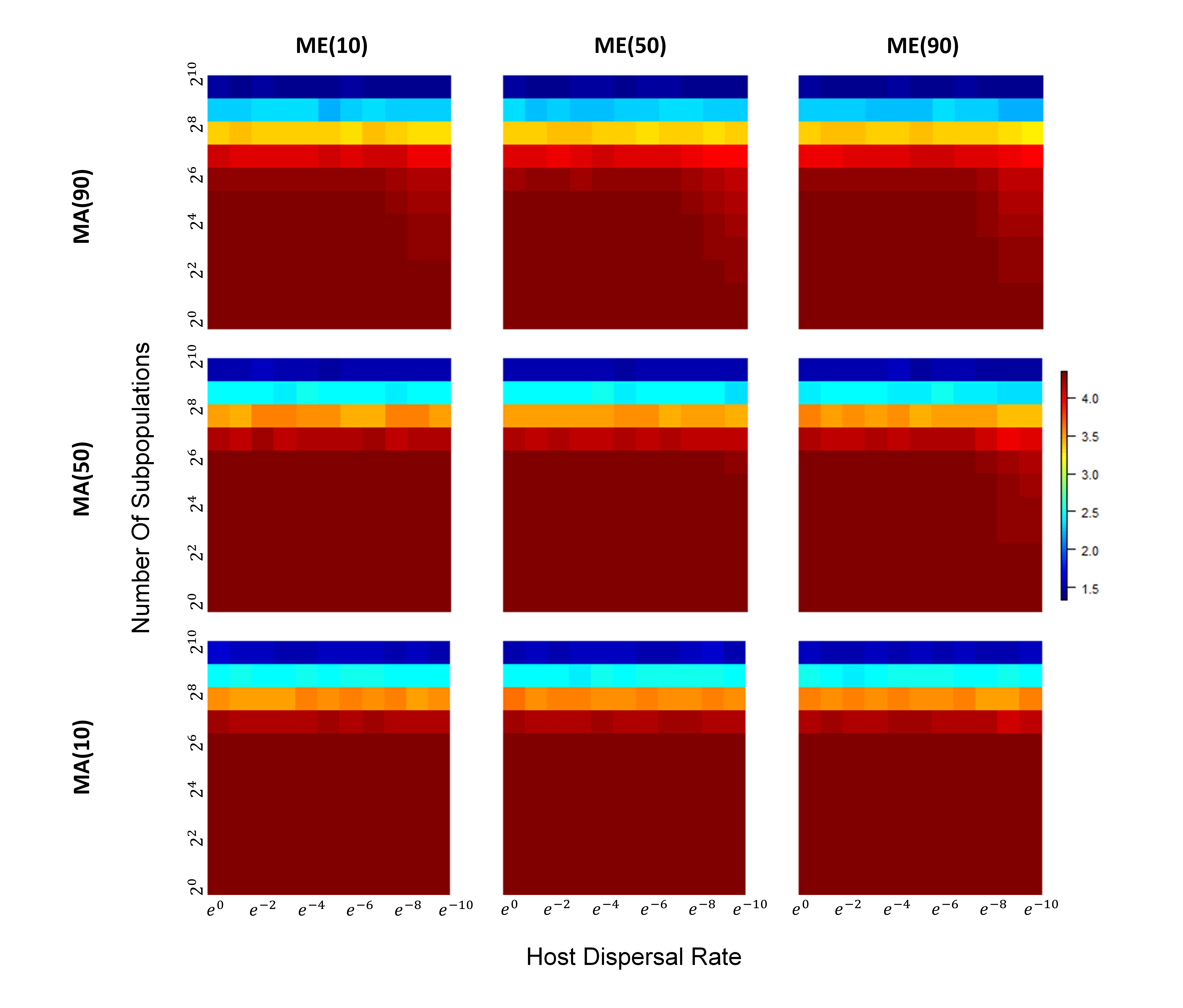

Supplement: Supplementary file 2 — Figure S2. Heatmaps of average γw-diversity across a range subpopulation sizes and host dispersal rates under different combinations of MAx and MEy. With a similar layout, all heatmaps are also plotted in the same way as those in Fig. 4 except that γw-diversity within subpopulations instead of α-diversity is measured. (TIF 1286 kb) [file 40168_2018_464_MOESM2_ESM.tif]

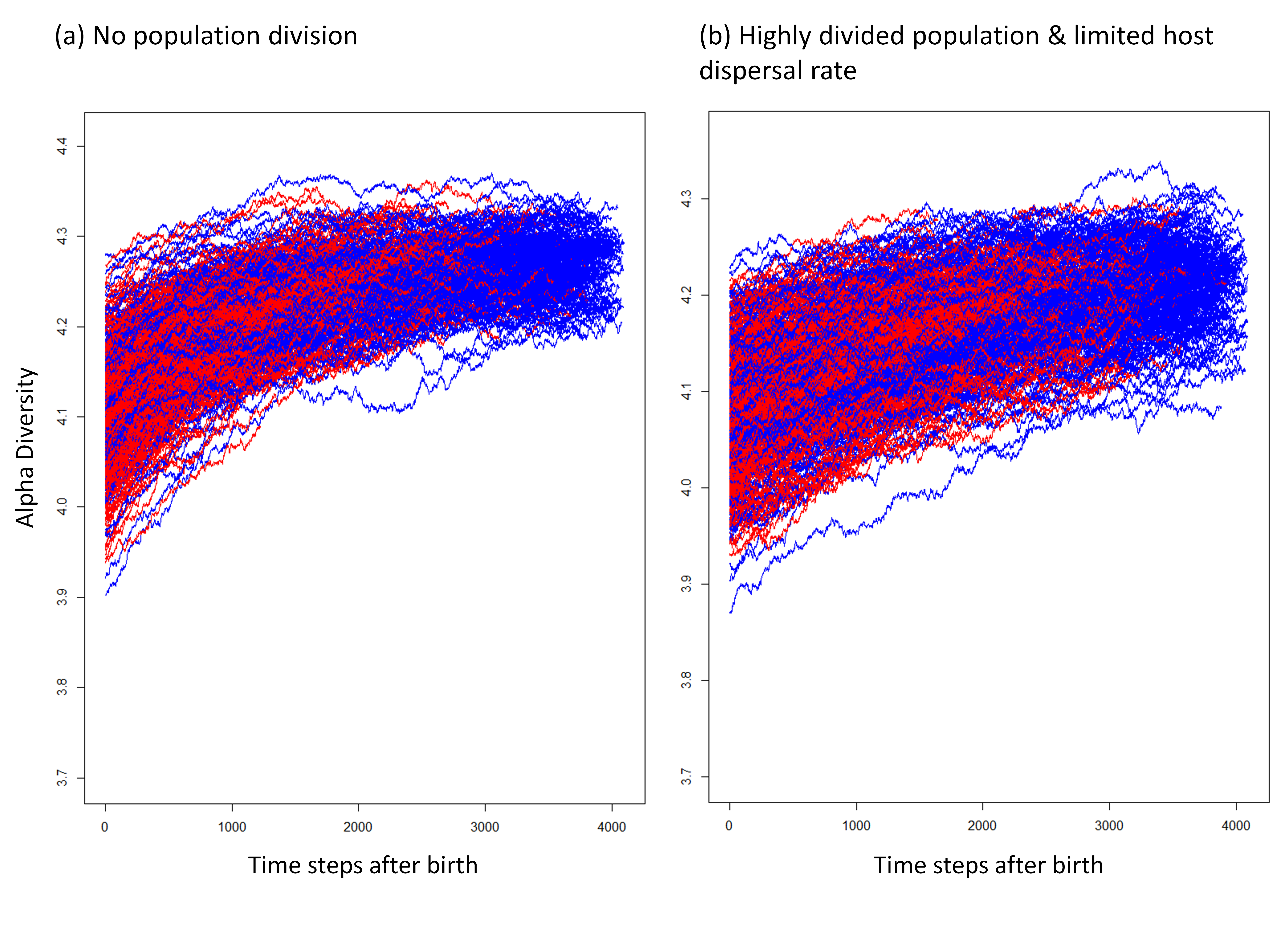

Supplement: Supplementary file 3 — Figure S3. α-diversity trace plots of individual microbiomes over host lifespan. Individual within-host diversity plots show under both structured population (1024 demes and host migration rate = e− 10) and unstructured population, individual α-diversity increases along time after birth. The blue lines represent hosts who are still alive before our simulation ends. The red lines represent hosts whose death events are observed by the end of our simulation. (TIF 5367 kb) [file 40168_2018_464_MOESM3_ESM.tif]
